# Fragranced consumer products: effects on asthmatic Australians

**DOI:** 10.1007/s11869-018-0560-x

**Published:** 2018-03-17

**Authors:** Anne Steinemann, Amanda J. Wheeler, Alexander Larcombe

**Affiliations:** 1 Department of Infrastructure Engineering, Melbourne School of Engineering, The University of Melbourne, Melbourne, VIC 3010 Australia; 2 College of Science and Engineering, James Cook University, Townsville, QLD 4811 Australia; 3 Climate, Atmospheric Sciences, and Physical Oceanography, Scripps Institution of Oceanography, University of California, San Diego, La Jolla, CA 92093 USA; 4 Menzies Institute for Medical Research, University of Tasmania, Hobart, TAS 7000 Australia; 5 Respiratory Environmental Health, Telethon Kids Institute, The University of Western Australia, Perth, WA 6008 Australia; 6 School of Public Health, Curtin University, Perth, WA 6102 Australia

**Keywords:** Fragranced consumer products, Indoor air quality, Exposure, Asthma, Air fresheners, Fragrance-free policy

## Abstract

Exposure to fragranced consumer products, such as air fresheners and cleaning supplies, is associated with adverse health effects such as asthma attacks, breathing difficulties, and migraine headaches. This study investigated the prevalence and types of health problems associated with exposure to fragranced products among asthmatic Australians. Nationally representative cross-sectional data were obtained in June 2016 with an online survey of adult Australians (*n* = 1098), of which 28.5% were medically diagnosed with asthma or an asthma-like condition. Nationally, 55.6% of asthmatics, and 23.9% of non-asthmatics, report adverse health effects after exposure to fragranced products. Specifically, 24.0% of asthmatics report an asthma attack. Moreover, 18.2% of asthmatics lost workdays or a job in the past year due to fragranced products in the workplace. Over 20% of asthmatics are unable to access public places and restrooms that use air fresheners. Exposure to fragranced products is associated with health problems, some potentially serious, in an estimated 2.2 million asthmatic adult Australians. Asthmatics were proportionately more affected than non-asthmatics (prevalence odds ratio 3.98; 95% confidence interval 3.01–5.24). Most asthmatics would prefer workplaces, healthcare facilities, and environments that are fragrance-free, which could help reduce adverse effects.

**Electronic supplementary material:**

The online version of this article (10.1007/s11869-018-0560-x) contains supplementary material, which is available to authorized users.

## Introduction

Exposure to fragranced consumer products, such as air fresheners, cleaning supplies, laundry detergents, perfumes, household items, and personal care products, has been associated with adverse human health effects (Steinemann 2009). Effects include headaches and migraines (Andress-Rothrock et al. 2010; Silva-Néto et al. 2013), contact dermatitis (Johansen 2003; Rastogi et al. 2007), infant diarrhea and earache (Farrow et al. 2003), reductions in lung and pulmonary function (Dales et al. 2013; Shim and Williams 1986), irritation of the airway mucosa (Elberling et al. 2005), and exacerbation of asthma symptoms (Kumar et al. 1995; Millqvist and Löwhagen 1996; Shim and Williams 1986; Weinberg et al. 2017).

In a study parallel to this one, Steinemann (2016) found that 34.7% of adult Americans reported health problems when exposed to fragranced products. Specifically, among Americans diagnosed with asthma or an asthma-like condition (26.8%), Steinemann (2017b) found that 64.3% report one or more types of adverse health effects, including respiratory problems (43.3%), migraine headaches (28.2%), and asthma attacks (27.9%), when exposed to fragranced consumer products. Another recent study showed that 3.8% of all work-related asthma cases reported in California between 1993 and 2012 were associated with fragranced product exposure, with the majority attributed to perfume or scented body products (Weinberg et al. 2017). Earlier studies found a higher prevalence of adverse health effects due to air freshener or deodorizer exposure among asthmatics (37.2%) than in the general population (20.5%), along with a higher rate of increase among asthmatics (7.5% compared with 3.0% over 3 years, 2002–2003 to 2005–2006) (Caress and Steinemann 2009).

Fragranced consumer products emit numerous volatile organic compounds (Steinemann 2015), and a single “fragrance” in a product is typically a mixture of several dozen to several hundred chemicals (Bickers et al. 2003). However, ingredients in fragranced consumer products do not need to be fully disclosed to the public on either the label or safety data sheet (Lunny et al. 2017). If any ingredients are disclosed, they typically represent fewer than 10% of all ingredients detected in product analyses (e.g., Uhde and Schulz 2015; Steinemann 2017c; Steinemann et al. 2011). For instance, Steinemann (2015) found more than 156 volatile organic compounds emitted from 37 fragranced consumer products, the most common being terpenes (e.g., limonene, alpha-pinene, and beta-pinene). However, of over 550 volatile ingredients emitted collectively, including hazardous air pollutants (e.g., formaldehyde), fewer than 3% were disclosed on the product labels, safety data sheets, or websites. Further, even the so-called green, organic, and natural fragranced products emitted hazardous air pollutants, similar to regular fragranced products (Steinemann 2015).

The primary objective of this study was to determine the prevalence and types of health and societal effects associated with exposure to fragranced products in the Australian population, with a focus on individuals with medically diagnosed asthma or an asthma-like condition. Steinemann (2017a) reports results from the general population survey of Australians, including both asthmatics and non-asthmatics, finding that 33% of Australians report adverse effects. This paper extends and enriches those results by investigating the effects specifically on the sub-population of asthmatics, and comparing them with non-asthmatics.

## Methods

Using an online national population survey, cross-sectional data were obtained on types of exposures to fragranced consumer products, associated effects on health, effects on work and quality of life, awareness of fragranced product ingredients and labeling, and preferences for fragrance-free environments. The survey contained 35 questions (outlined in the Supplementary Material), each on its own page, with multiple choice and open format response categories. Categories of fragranced products evaluated in the survey included air fresheners and deodorizers, personal care products, cleaning supplies, laundry products, household products, and fragrances.

Drawing upon a large panel of over 200,000 Australian participants held by Survey Sampling International (SSI), the survey was conducted in June 2016 with 1098 adult (ages 18 to 65) Australians, representative of age, gender, and region (confidence limit = 95%, margin of error = 3%). SSI is a survey research company and online panel provider. Participant recruitment followed a randomized process, and all responses were anonymous. Survey completion time was approximately 10 min. No personal information was linked to the survey results, and data were stored on password-protected computers. Additional details on survey data and methodology, including the Checklist for Reporting Results of Internet E-Surveys (CHERRIES, Eysenbach 2004), are provided in the Supplementary Material.

Out of 1098 participants, 313 (28.5%) answered “yes” to being medically diagnosed by a doctor or healthcare professional as having asthma or an asthma-like condition. For brevity, these participants will hereafter be referred to as “asthmatics,” and the other 785 respondents (71.5%) will be referred to as “non-asthmatics.” Demographics of the survey respondents are shown in Table 1. Results are summarized below in four main sections: health problems associated with exposure, effects of exposure on work and quality of life, awareness of product emissions, and preferences for fragrance-free environments.

**Table 1:** Study participant demographics

	Asthmatic (*n*, %)	Non-asthmatic (*n*, %)
Total	313, 100.0%	785, 100.0%
All Males	143, 45.7%	400, 51.0%
All Females	170, 54.3%	385, 49.0%
Male 18–24	16, 5.1%	54, 6.9%
Male 25–34	34, 10.9%	75, 9.6%
Male 35–44	33, 10.5%	86, 11.0%
Male 45–54	34, 10.9%	92, 11.7%
Male 55–65	26, 8.3%	93, 11.8%
Female 18–24	26, 8.3%	60, 7.6%
Female 25–34	35, 11.2%	95, 12.1%
Female 35–44	42, 13.4%	95, 12.1%
Female 45–54	41, 13.1%	74, 9.4%
Female 55–65	26, 8.3%	61, 7.8%
Australian Capital Territory	6, 1.9%	14, 1.8%
New South Wales	98, 31.3%	262, 33.4%
Northern Territory	2, 0.6%	5, 0.6%
Queensland	66, 21.1%	151, 19.2%
South Australia	25, 8.0%	60, 7.6%
Tasmania	7, 2.2%	18, 2.3%
Victoria	77, 24.6%	201, 25.6%
Western Australia	32, 10.2%	74, 9.4%

### Statistical analyses

Descriptive statistics and cross tabulations, calculated using GraphPad Prism v6.07 (GraphPad Software Inc.), determined the prevalence of outcomes by different sub-populations and demographics. Chi-squared (*χ*^2^) analyses with Yates’ correction, also calculated using GraphPad Prism v6.07, compared the proportion of asthmatics with the proportion of non-asthmatics who answered “yes” to survey questions to determine whether a statistically significant difference exists. Prevalence odds ratios (PORs) were calculated to measure the strength of the association between reported health effects and asthmatics versus non-asthmatics to determine whether asthmatics are proportionally more affected than non-asthmatics. All *χ*^2^ and prevalence odds ratio analyses were conducted using a 95% confidence level or confidence intervals. Further details on methodology and survey data are provided in the Supplementary Material.

## Results

Key findings of this study are that exposure to fragranced products is ubiquitous in Australian society, that it is associated with a range of adverse health effects in 33% of the general population, and that these adverse health outcomes affect asthmatics (55.6%) more than non-asthmatics (23.9%) (POR 3.98; 95% CI 3.01–5.24). Further, in each category of adverse health effect investigated in this study, asthmatics are proportionately more affected than non-asthmatics (Table 2).

**Table 2:** Prevalence and types of health problems reported by individuals who experienced adverse health effects associated with exposure to fragranced products

	Asthmatic		Non-asthmatic		POR (95% CI)
	Prevalence (*n*)	Percent	Prevalence (*n*)	Percent	
Respiratory problems	106	33.9	77	9.8	4.71 (3.38–6.56)
Mucosal symptoms	83	26.5	71	9.0	3.63 (2.56–5.15)
Skin problems	52	16.6	52	6.6	2.81 (1.86–4.23)
Migraine headaches	53	16.9	57	7.3	2.60 (1.74–3.88)
Asthma attacks	75	24.0	8	1.0	30.61 (14.55–64.36)
Neurological problems	28	8.9	21	2.7	3.57 (1.98–6.40)
Cognitive problems	27	8.6	18	2.3	4.02 (2.18–7.42)
Gastrointestinal problems	21	6.7	15	1.9	3.69 (1.88–7.26)
Cardiovascular problems	21	6.7	12	1.5	4.63 (2.25–9.54)
Immune system problems	24	7.7	12	1.5	5.35 (2.64–10.84)
Musculoskeletal problems	18	5.8	11	1.4	4.29 (2.00–9.20)
Other	5	1.6	16	2.0	0.78 (0.28–2.15)
Total	174	55.6	188	23.9	3.98 (3.01–5.24)

### Health problems associated with exposure

Virtually all respondents are exposed to fragranced products at least once a week through their own use (99% for asthmatics, 97.6% for non-asthmatics), others’ use (92.3% for asthmatics, 86.4% for non-asthmatics), or both (99.7% for asthmatics, 98.1% for non-asthmatics).

More than half (55.6%) of asthmatics reported health problems from exposure to one or more types of fragranced products, compared with approximately one quarter (23.9%) of non-asthmatics (*χ*^2^ = (1, *N* = 1098) = 97.13, *p* < 0.001) (Table 2 and Supplementary Material). Significantly, 24.0% of asthmatics reported that exposure to a fragranced product was associated with an asthma attack.

Both asthmatics and non-asthmatics reported health problems when exposed to air fresheners or deodorizers (33.9% for asthmatics and 9.4% for non-asthmatics), scented laundry products coming from a dryer vent (12.1% and 3.7%, respectively), being in a room after it has been cleaned with scented products (30.7% and 9.2%, respectively), and being near someone who is wearing a fragranced product (36.1% and 12.7%, respectively), with asthmatics proportionately more affected than non-asthmatics (POR 3.98; 95% CI 3.01–5.24) (see Table 3).

**Table 3:** Percentage of individuals who experienced adverse health effects associated with four types of exposure to fragranced products. AF = air fresheners or deodorizers, LP = scent of laundry products coming from a dryer vent, CP = being in a room after it has been cleaned with scented products, FP = being near someone wearing a fragranced product

	Asthmatic				Non-asthmatic			
	AF	LP	CP	FP	AF	LP	CP	FP
Respiratory problems	20.8	5.1	16.6	18.2	4.5	1.5	3.8	4.3
Mucosal symptoms	13.7	4.2	13.1	15.7	3.2	0.5	3.2	4.8
Skin problems	9.3	4.8	5.1	4.2	3.1	1.0	1.9	0.8
Migraine headaches	9.9	2.2	7.0	7.3	1.9	0.9	2.0	3.9
Asthma attacks	14.1	4.5	8.3	10.5	0.6	0.1	0.1	0.5
Neurological problems	5.8	2.6	4.2	4.8	0.8	0.0	0.5	1.4
Cognitive problems	4.5	2.6	4.2	3.5	0.9	0.4	0.9	0.8
Gastrointestinal problems	3.8	3.2	4.2	2.2	0.5	0.4	0.4	1.1
Cardiovascular problems	4.2	4.2	3.8	2.9	1.0	0.3	0.3	0.5
Immune system problems	4.2	5.1	4.2	3.8	0.9	0.5	0.6	0.3
Musculoskeletal problems	3.5	2.9	2.6	3.5	0.9	0.1	0.4	0.3
Other	0.0	0.0	0.6	0.6	0.8	0.3	0.6	0.9
Total	33.9	12.1	30.7	36.1	9.4	3.7	9.2	12.7

Moreover, asthmatic Australians were more likely to report health problems for each of the 11 categories of adverse health effects investigated in the survey (*χ*^2^ = (1, *N* = 1098) > 14.82, *p* < 0.001 in all cases) (see Table 2). Respiratory problems (e.g., difficulty breathing, coughing, or shortness of breath) and mucosal symptoms (e.g., watery or red eyes, nasal congestion, or sneezing) were the most frequently reported health problems for both asthmatics (33.9% and 26.5%, respectively) and non-asthmatics (9.8% and 9.0%, respectively).

### Effects of exposure on work and quality of life

Fragranced products can also restrict access in society and impose costs due to their potential to adversely affect health. The survey found 21.4% of asthmatics and 7.6% of non-asthmatics are unable or reluctant to use the restrooms in a public place, because of the presence of an air freshener, deodorizer, or scented product (*χ*^2^ = (1, *N* = 1098) = 40.1, *p* < 0.0001). In addition, 20.8% of asthmatics and 6.1% of non-asthmatics are unable or reluctant to wash their hands with soap in a public place if the soap is fragranced (*χ*^2^ = (1, *N* = 1098) = 50.46, *p* < 0.0001). Further, 31.9% of asthmatics and 8.3% of non-asthmatics had been prevented from going to some place, because they would be exposed to a fragranced product that would make them sick (*χ*^2^ = (1, *N* = 1098) = 96.33, *p* < 0.0001). Upon entering a business where they could smell air fresheners or a fragranced product, 31.0% of asthmatics and 11.0% of non-asthmatics wanted to leave as quickly as possible (*χ*^2^ = (1, *N* = 1014) = 63.24, *p* < 0.0001). In the workplace, 18.2% of asthmatics and 3.6% of non-asthmatics have lost workdays or a job, in the past 12 months, due to exposure to fragranced products in their work environment (*χ*^2^ = (1, *N* = 1098) = 65.16, *p* < 0.001). Thus, in each of these exposure situations, asthmatics were significantly more likely to report adverse effects than non-asthmatics.

### Awareness of product emissions

Overall, the majority of asthmatics and non-asthmatics were unaware of potentially hazardous chemicals emitted from fragranced products, even ones called green or organic, and would not continue to use a fragranced product if they knew it emitted these pollutants. Nearly half of asthmatics (42.2%) and non-asthmatics (49.2%) were not aware that a “fragrance” in a product is a mixture of several dozen to several hundred chemicals (*χ*^2^ = (1, *N* = 1098) = 4.123, *p* = 0.042). Most asthmatics (67.1%) and non-asthmatics (69.2%) were not aware that fragrance chemicals do not need to be fully disclosed on the product label or material safety data sheet (*χ*^2^ = (1, *N* = 1098) = 0.3577, *p* = 0.550). Similarly, most asthmatics (70.3%) and non-asthmatics (75.0%) did not know that fragranced products, even those called natural, green or organic, typically emit hazardous air pollutants (*χ*^2^ = (1, *N* = 1098) = 2.358, *p* = 0.125). Importantly, more than half of asthmatics (60.1%) and of non-asthmatics (54.8%) would not continue to use a fragranced product if they knew it emitted hazardous air pollutants (*χ*^2^ = (1, *N* = 1098) = 2.332, *p* = 0.127). Thus, asthmatics and non-asthmatics were similarly unaware of fragranced product emissions of hazardous air pollutants and would similarly cease use of a fragranced product if they were aware of such emissions.

### Preferences for fragrance-free environments

Fragrance-free environments received widespread support from both asthmatics and non-asthmatics (Table 4). More than half of asthmatics (50.5%) and nearly half of non-asthmatics (39.7%) would support a fragrance-free policy in the workplace, representing almost twice as many that would not support a policy (*χ*^2^ = (1, *N* = 714) = 6.921, *p* = 0.008). Nearly half of both asthmatics (50.2%) and non-asthmatics (40.4%) would prefer that healthcare facilities and healthcare professionals were fragrance-free (*χ*^2^ = (1, *N* = 751) = 1.779, *p* = 0.182). A majority of asthmatics (62.6%) and non-asthmatics (55.8%) would prefer an airplane without scented air pumped through the passenger cabin, representing over three times as many that would prefer unscented air to scented air (*χ*^2^ = (1, *N* = 813) = 0.082, *p* = 0.773). A majority of asthmatics (60.1%) and non-asthmatics (53.8%) would prefer a hotel without scented air, representing over twice as many that would prefer unscented air to scented air (*χ*^2^ = (1, *N* = 714) = 0.124, *p* = 0.724).

**Table 4:** Percentage of individuals who prefer fragrance-free environments

	Asthmatic				Non-asthmatic			
	Yes	No	Neutral/not sure	Decline to answer	Yes	No	Neutral/not sure	Decline to answer
Fragrance-free workplaces	50.5%	18.5%	31.0%	0.0%	39.7%	23.7%	36.2%	0.4%
Fragrance-free healthcare facilities and healthcare professionals	50.2%	24.9%	24.6%	0.3%	40.4%	25.4%	33.8%	0.5%
Airplanes without scented air	62.6%	18.5%	18.8%	0.0%	55.8%	15.4%	28.5%	0.3%
Hotels without scented air	60.1%	23.3%	16.6%	0.0%	53.8%	22.4%	23.6%	0.3%

## Discussion

Results revealed that nearly all asthmatics used a fragranced product (99.0%) or were involuntarily exposed to others’ use of fragranced products (92.3%), or both (99.7%), at least once a week. Thus, the potential for exposure is widespread, especially from involuntary exposure. These results are similar to a nationally representative US population study (Steinemann 2016) showing widespread exposure to fragranced products from own use (98.3%), others’ use (92.1%), or both (99.1%) (*χ*^2^ = (1, *N* = 618) = 0.003, *p* = 0.955).

An individual’s ability to avoid exposure to fragranced products can also depend on their awareness of product emissions. As prior work has demonstrated (Steinemann 2015; Lunny et al. 2017), ingredients in fragranced products are not fully disclosed, making it nearly impossible for the public to make informed decisions. This problem is exacerbated because even if products did list all their ingredients, we lack information on which chemicals, mixtures, or reaction products could be associated with the reported adverse effects. In this study, a majority of asthmatics and non-asthmatics were unaware of the potentially hazardous ingredients in fragranced products, and would not use a fragranced product if they knew it emitted hazardous air pollutants. This indicates that many Australians may be using fragranced products without their awareness of exposures. However, products without a fragrance are widely available, and offer a practical option to reduce risks for people adversely affected by fragranced products.

Our results indicate that over half of asthmatics (55.6%), and almost one quarter of non-asthmatics (23.9%), reported one or more types of adverse health effects associated with exposure to one or more types of fragranced products. Asthmatics were more likely to experience adverse health effects from fragranced products (POR 3.98; 95% CI 3.01–5.24), regardless of the type of adverse effect (Table 2). Commonly, asthmatics experienced respiratory problems (POR 4.71; 95% CI 3.38–6.56) and mucosal symptoms (POR 3.63; 95% CI 2.56–5.15) after exposure to a fragranced product. Similarly, among Americans, asthmatics were more likely to experience adverse health effects from fragranced products than non-asthmatics (POR 5.76; 95% CI 4.34–7.64) (Steinemann 2017a). Also, in two nationally representative studies in the USA, Caress and Steinemann (2009) found 29.7 and 37.2% of asthmatics (in 2002–2003 and 2005–2006, respectively) reported health problems from air fresheners and deodorizers, compared with 33.9% of asthmatics in our study.

In addition to direct impacts on health, fragranced products can also restrict an individual’s access in society, affect their lifestyle, and have economic and workplace implications. Our findings indicate that 18.2% of asthmatics face economic implications through loss of workdays or a job, in the past year, due to fragranced product exposure in the workplace. Over twice as many asthmatics and non-asthmatics would support a fragrance-free policy in the workplace (50.5 and 39.7%, respectively), compared with those that would not (18.5 and 23.7%, respectively).

### Study strengths and limitations

Our survey population is representative of the Australian population (1098 respondents, 95% confidence level with a 3% margin of error). The 1098 respondents were randomly recruited from a large Web-based panel (over 200,000 potential participants). The proportion of respondents who stated they had doctor or healthcare professional diagnosed asthma (16.0%) is similar to the proportion (19.0%) reported in the 2007–2008 National Health Survey (AIHW 2011). Our survey questions were based on a study previously conducted and published (Steinemann 2017b). Study limitations include its cross-sectional design, being restricted to adults (18–65 years), and reliance on self-reported data. The cross-sectional design means that we are unable to infer temporal associations between exposures and outcomes. Restricting respondents to adults between 18 and 65 years of age excludes data on effects of fragranced products on children and the elderly. Self-reports are commonly used in survey-based research; however, respondent data were not externally verified. Our lists of potential health effects were not exhaustive; however, only 1.6% of asthmatics and 2.0% of non-asthmatics reported health effects “other” than those included in our lists, suggesting that primary effects were likely captured. Finally, we lack data on the severity or frequency of adverse health outcomes, such as whether exposure to a single fragranced product was associated with a single or multiple health outcomes.

In our survey, 28.5% of respondents answered “yes” to having either doctor or healthcare professional diagnosed asthma (16.0%), or an asthma-like condition (13.8%), or both. Thus, the 55.6% of “asthmatics” in our study who report adverse health effects due to fragranced product exposure would represent over 2.2 million adult Australians (ABS 2016). Combining this with the 23.9% of “non-asthmatics” (71.5%) who also report adverse health effects would represent over 4.5 million adult Australians affected adversely by fragranced consumer products.

## Conclusions

Results from this study show that voluntary and involuntary exposure to fragranced products is widespread in Australian society, that exposure is associated with a range of potentially serious and adverse health effects, and that these effects are more common in people with asthma or an asthma-like condition. In addition to the adverse health consequences, exposure to fragranced products imposes significant adverse impacts on workplace productivity and quality of life, including the ability to access public places such as restrooms. A straightforward approach to reduce undesirable effects would be to reduce or avoid use of fragranced products, especially in public places that would impose involuntary risks, and to implement fragrance-free policies in workplaces, healthcare facilities, and other environments.

## Electronic supplementary material

ESM 1 (PDF 267 kb)

ESM 2 (PDF 55 kb)
