# Supplementary material for: Fragranced consumer products: effects on asthmatic Australians
Source: Air Qual Atmos Health. 2018 Mar 17;11(4):365–71. doi: 10.1007/s11869-018-0560-x (PMC5954056; doi:10.1007/s11869-018-0560-x)
Supplement: Supplementary file 1 — (PDF 267 kb) [file 11869_2018_560_MOESM1_ESM.pdf]

Supplementary Material 2 - Survey results

Table 1

Asthmatic, Non-Asthmatic, and General Population proportions

	Asthmatic	Non-Asthmatic	GenPop
**Total**	313	785	1098
	100.00%	100.00%	100.00%

Table 2

What is your gender?

	Asthmatic	Non-Asthmatic	GenPop
Total	313	785	1098
	100.00%	100.00%	100.00%
Male	143	400	543
	45.70%	51.00%	49.50%
Female	170	385	555
	54.30%	49.00%	50.50%
Other	-	-	-
	-	-	-
SUM	313	785	1098
	100.00%	100.00%	100.00%

Table 3

What is your age?

	Asthmatic	Non-Asthmatic	GenPop
**Total**	313	785	1098
	100.00%	100.00%	100.00%
**18-24 (21)**	42	114	156
	13.40%	14.50%	14.20%
**25-34 (29.5)**	69	170	239
	22.00%	21.70%	21.80%
**35-44 (39.5)**	75	181	256
	24.00%	23.10%	23.30%
**45-54 (49.5)**	75	166	241
	24.00%	21.10%	21.90%
**55-65 (60)**	52	154	206
	16.60%	19.60%	18.80%
**65 (65)**	-	-	-
	-	-	-
**Mean**	40.62	40.78	40.74
**Standard Deviation**	12.69	13.17	13.03
**Standard Error**	0.72	0.47	0.39
**SUM**	313	785	1098
	100.00%	100.00%	100.00%

Table 4

AUSTRALIA Region.

	Asthmatic	Non-Asthmatic	GenPop
**Total**	313	785	1098
	100.00%	100.00%	100.00%
**Australian Capital Territory**	6	14	20
	1.90%	1.80%	1.80%
**New South Wales**	98	262	360
	31.30%	33.40%	32.80%
**Northern Territory**	2	5	7
	0.60%	0.60%	0.60%
**Queensland**	66	151	217
	21.10%	19.20%	19.80%
**South Australia**	25	60	85
	8.00%	7.60%	7.70%
**Tasmania**	7	18	25
	2.20%	2.30%	2.30%
**Victoria**	77	201	278
	24.60%	25.60%	25.30%
**Western Australia**	32	74	106
	10.20%	9.40%	9.70%
**SUM**	313	785	1098
	100.00%	100.00%	100.00%

Table 5

Q1. Which fragranced products are you exposed to, at least once a week, from your own use?

	Asthmatic	Non-Asthmatic	GenPop
**Total**	313	785	1098
	100.00%	100.00%	100.00%
**Air fresheners and deodorizers (e.g., sprays, solids, oils, disks)**	220	513	733
	70.30%	65.40%	66.80%
**Personal care products (e.g., soaps, hand sanitizer, lotions, deodorant, sunscreen, shampoos)**	293	713	1006
	93.60%	90.80%	91.60%
**Cleaning supplies (e.g., all-purpose cleaners, disinfectants, and dishwashing soap)**	263	650	913
	84.00%	82.80%	83.20%
**Laundry products (e.g., detergents, fabric softeners, dryer sheets)**	273	653	926
	87.20%	83.20%	84.30%
**Household products (e.g., scented candles, toilet paper, trash bags, baby products)**	247	600	847
	78.90%	76.40%	77.10%
**Fragrance (e.g., perfume, cologne, after-shave)**	223	541	764
	71.20%	68.90%	69.60%
**Other**	5	20	25
	1.60%	2.50%	2.30%
**None**	3	19	22
	1.00%	2.40%	2.00%
**SUM**	1527	3709	5236
	487.90%	472.50%	476.90%

Table 6

Q1a. Which fragranced products are you exposed to, at least once a week, from others' use?

	Asthmatic	Non-Asthmatic	GenPop
**Total**	313	785	1098
	100.00%	100.00%	100.00%
**Air fresheners and deodorizers (e.g., sprays, solids, oils, disks)**	174	384	558
	55.60%	48.90%	50.80%
**Personal care products (e.g., soaps, hand sanitizer, lotions, deodorant, sunscreen, shampoos)**	206	469	675
	65.80%	59.70%	61.50%
**Cleaning supplies (e.g., all-purpose cleaners, disinfectants, and dishwashing soap)**	169	388	557
	54.00%	49.40%	50.70%
**Laundry products (e.g., detergents, fabric softeners, dryer sheets)**	140	346	486
	44.70%	44.10%	44.30%
**Household products (e.g., scented candles, toilet paper, trash bags, baby products)**	162	383	545
	51.80%	48.80%	49.60%
**Fragrance (e.g., perfume, cologne, after-shave)**	237	507	744
	75.70%	64.60%	67.80%
**Other**	6	14	20
	1.90%	1.80%	1.80%
**None**	24	107	131
	7.70%	13.60%	11.90%
**SUM**	1118	2598	3716
	357.20%	331.00%	338.40%

Table 7

Q2. Do you experience any health problems when exposed to air fresheners or deodorizers?

	Asthmatic	Non-Asthmatic	GenPop
**Total**	313	785	1098
	100.00%	100.00%	100.00%
**Yes**	106	74	180
	33.90%	9.40%	16.40%
**No**	164	596	760
	52.40%	75.90%	69.20%
**Don't know/not sure**	43	114	157
	13.70%	14.50%	14.30%
**Decline to answer**	-	1	1
	-	0.10%	0.10%
**SUM**	313	785	1098
	100.00%	100.00%	100.00%

Table 8

BA. Which of the following health problems do you experience?

Base: Respondents who experienced below health problems when exposed to air fresheners or deodorizers

	Asthmatic	Non-Asthmatic	GenPop
**Total**	106	74	180
	100.00%	100.00%	100.00%
**Migraine headaches**	31	15	46
	29.20%	20.30%	25.60%
**Asthma attacks**	44	5	49
	41.50%	6.80%	27.20%
**Neurological problems (e.g., dizziness, seizures, head pain, fainting, loss of coordination)**	18	6	24
	17.00%	8.10%	13.30%
**Respiratory problems (e.g., difficulty breathing, coughing, shortness of breath)**	65	35	100
	61.30%	47.30%	55.60%
**Skin problems (e.g., rashes, hives, red skin, tingling skin, dermatitis)**	29	24	53
	27.40%	32.40%	29.40%
**Cognitive problems (e.g., difficulties thinking, concentrating, or remembering)**	14	7	21
	13.20%	9.50%	11.70%
**Mucosal symptoms (e.g., watery or red eyes, nasal congestion, sneezing)**	43	25	68
	40.60%	33.80%	37.80%
**Immune system problems (e.g., swollen lymph glands, fever, fatigue)**	13	7	20
	12.30%	9.50%	11.10%
**Gastrointestinal problems (e.g., nausea, bloating, cramping, diarrhea)**	12	4	16
	11.30%	5.40%	8.90%
**Cardiovascular problems (e.g., fast or irregular heartbeat, jitteriness, chest discomfort)**	13	8	21
	12.30%	10.80%	11.70%
**Musculoskeletal problems (e.g., muscle or joint pain, cramps, weakness)**	11	7	18
	10.40%	9.50%	10.00%
**Other**	-	6	6
	-	8.10%	3.30%
**SUM**	293	149	442
	276.40%	201.40%	245.60%

Table 9

Q3. Do you experience any health problems from the scent of laundry products coming from a dryer vent?

	Asthmatic	Non-Asthmatic	GenPop
**Total**	313	785	1098
	100.00%	100.00%	100.00%
**Yes**	38	29	67
	12.10%	3.70%	6.10%
**No**	231	678	909
	73.80%	86.40%	82.80%
**Don't know/not sure**	44	76	120
	14.10%	9.70%	10.90%
**Decline to answer**	-	2	2
	-	0.30%	0.20%
**SUM**	313	785	1098
	100.00%	100.00%	100.00%

Table 10

BA. Which of the following health problems do you experience?

Base: Respondents who experienced below health problems from the scent of laundry products coming from a dryer vent

	Asthmatic	Non-Asthmatic	GenPop
**Total**	38	29	67
	100.00%	100.00%	100.00%
**Migraine headaches**	7	7	14
	18.40%	24.10%	20.90%
**Asthma attacks**	14	1	15
	36.80%	3.40%	22.40%
**Neurological problems (e.g., dizziness, seizures,**	8	-	8
**head pain, fainting, loss of coordination)**	21.10%	-	11.90%
**Respiratory problems (e.g., difficulty breathing,**	16	12	28
**coughing, shortness of breath)**	42.10%	41.40%	41.80%
**Skin problems (e.g., rashes, hives, red skin,**	15	8	23
**tingling skin, dermatitis)**	39.50%	27.60%	34.30%
**Cognitive problems (e.g., difficulties thinking,**	8	3	11
**concentrating, or remembering)**	21.10%	10.30%	16.40%
**Mucosal symptoms (e.g., watery or red eyes, nasal**	13	4	17
**congestion, sneezing)**	34.20%	13.80%	25.40%
**Immune system problems (e.g., swollen lymph**	16	4	20
**glands, fever, fatigue)**	42.10%	13.80%	29.90%
**Gastrointestinal problems (e.g., nausea, bloating,**	10	3	13
**cramping, diarrhea)**	26.30%	10.30%	19.40%
**Cardiovascular problems (e.g., fast or irregular**	13	2	15
**heartbeat, jitteriness, chest discomfort)**	34.20%	6.90%	22.40%
**Musculoskeletal problems (e.g., muscle or joint**	9	1	10
**pain, cramps, weakness)**	23.70%	3.40%	14.90%
**Other**	-	2	2
	-	6.90%	3.00%
**SUM**	129	47	176
	339.50%	162.10%	262.70%

Table 11

Q4. Do you experience any health problems from being in a room after it has been cleaned with scented products?

	Asthmatic	Non-Asthmatic	GenPop
**Total**	313	785	1098
	100.00%	100.00%	100.00%
**Yes**	96	72	168
	30.70%	9.20%	15.30%
**No**	192	625	817
	61.30%	79.60%	74.40%
**Don't know/not sure**	25	85	110
	8.00%	10.80%	10.00%
**Decline to answer**	-	3	3
	-	0.40%	0.30%
**SUM**	313	785	1098
	100.00%	100.00%	100.00%

Table 12

BA. Which of the following health problems do you experience?

Base: Respondents who experienced below health problems from being in a room after it has been cleaned with scented products

	Asthmatic	Non-Asthmatic	GenPop
**Total**	96	72	168
	100.00%	100.00%	100.00%
**Migraine headaches**	22	16	38
	22.90%	22.20%	22.60%
**Asthma attacks**	26	1	27
	27.10%	1.40%	16.10%
**Neurological problems (e.g., dizziness, seizures,**	13	4	17
**head pain, fainting, loss of coordination)**	13.50%	5.60%	10.10%
**Respiratory problems (e.g., difficulty breathing,**	52	30	82
**coughing, shortness of breath)**	54.20%	41.70%	48.80%
**Skin problems (e.g., rashes, hives, red skin,**	16	15	31
**tingling skin, dermatitis)**	16.70%	20.80%	18.50%
**Cognitive problems (e.g., difficulties thinking,**	13	7	20
**concentrating, or remembering)**	13.50%	9.70%	11.90%
**Mucosal symptoms (e.g., watery or red eyes, nasal**	41	25	66
**congestion, sneezing)**	42.70%	34.70%	39.30%
**Immune system problems (e.g., swollen lymph**	13	5	18
**glands, fever, fatigue)**	13.50%	6.90%	10.70%
**Gastrointestinal problems (e.g., nausea, bloating,**	13	3	16
**cramping, diarrhea)**	13.50%	4.20%	9.50%
**Cardiovascular problems (e.g., fast or irregular**	12	2	14
**heartbeat, jitteriness, chest discomfort)**	12.50%	2.80%	8.30%
**Musculoskeletal problems (e.g., muscle or joint**	8	3	11
**pain, cramps, weakness)**	8.30%	4.20%	6.50%
**Other**	2	5	7
	2.10%	6.90%	4.20%
**SUM**	231	116	347
	240.60%	161.10%	206.50%

Table 13

Q5. Do you experience any health problems from being near someone who is wearing a fragranced product?

	Asthmatic	Non-Asthmatic	GenPop
**Total**	313	785	1098
	100.00%	100.00%	100.00%
**Yes**	113	100	213
	36.10%	12.70%	19.40%
**No**	171	622	793
	54.60%	79.20%	72.20%
**Don't know/not sure**	28	62	90
	8.90%	7.90%	8.20%
**Decline to answer**	1	1	2
	0.30%	0.10%	0.20%
**SUM**	313	785	1098
	100.00%	100.00%	100.00%

Table 14

BA. Which of the following health problems do you experience?

Base: Respondents who experienced below health problems from being near someone who is wearing a fragranced product

	Asthmatic	Non-Asthmatic	GenPop
**Total**	113	100	213
	100.00%	100.00%	100.00%
**Migraine headaches**	23	31	54
	20.40%	31.00%	25.40%
**Asthma attacks**	33	4	37
	29.20%	4.00%	17.40%
**Neurological problems (e.g., dizziness, seizures,**	15	11	26
**head pain, fainting, loss of coordination)**	13.30%	11.00%	12.20%
**Respiratory problems (e.g., difficulty breathing,**	57	34	91
**coughing, shortness of breath)**	50.40%	34.00%	42.70%
**Skin problems (e.g., rashes, hives, red skin,**	13	6	19
**tingling skin, dermatitis)**	11.50%	6.00%	8.90%
**Cognitive problems (e.g., difficulties thinking,**	11	6	17
**concentrating, or remembering)**	9.70%	6.00%	8.00%
**Mucosal symptoms (e.g., watery or red eyes, nasal**	49	38	87
**congestion, sneezing)**	43.40%	38.00%	40.80%
**Immune system problems (e.g., swollen lymph**	12	2	14
**glands, fever, fatigue)**	10.60%	2.00%	6.60%
**Gastrointestinal problems (e.g., nausea, bloating,**	7	9	16
**cramping, diarrhea)**	6.20%	9.00%	7.50%
**Cardiovascular problems (e.g., fast or irregular**	9	4	13
**heartbeat, jitteriness, chest discomfort)**	8.00%	4.00%	6.10%
**Musculoskeletal problems (e.g., muscle or joint**	11	2	13
**pain, cramps, weakness)**	9.70%	2.00%	6.10%
**Other**	2	7	9
	1.80%	7.00%	4.20%
**SUM**	242	154	396
	214.20%	154.00%	185.90%

Table 15

Q6. In general, do you experience any health problems from exposure to any type of fragranced product?

	Asthmatic	Non-Asthmatic	GenPop
**Total**	313	785	1098
	100.00%	100.00%	100.00%
**Yes**	124	99	223
	39.60%	12.60%	20.30%
**No**	163	615	778
	52.10%	78.30%	70.90%
**Don't know/not sure**	26	70	96
	8.30%	8.90%	8.70%
**Decline to answer**	-	1	1
	-	0.10%	0.10%
**SUM**	313	785	1098
	100.00%	100.00%	100.00%

Table 16

BA. Which of the following health problems do you experience?

Base: Respondents who experienced below health problems from exposure to any type of fragranced product

	Asthmatic	Non-Asthmatic	GenPop
**Total**	124	99	223
	100.00%	100.00%	100.00%
**Migraine headaches**	27	24	51
	21.80%	24.20%	22.90%
**Asthma attacks**	39	1	40
	31.50%	1.00%	17.90%
**Neurological problems (e.g., dizziness, seizures,**	14	8	22
**head pain, fainting, loss of coordination)**	11.30%	8.10%	9.90%
**Respiratory problems (e.g., difficulty breathing,**	72	38	110
**coughing, shortness of breath)**	58.10%	38.40%	49.30%
**Skin problems (e.g., rashes, hives, red skin,**	30	24	54
**tingling skin, dermatitis)**	24.20%	24.20%	24.20%
**Cognitive problems (e.g., difficulties thinking,**	11	8	19
**concentrating, or remembering)**	8.90%	8.10%	8.50%
**Mucosal symptoms (e.g., watery or red eyes, nasal**	48	35	83
**congestion, sneezing)**	38.70%	35.40%	37.20%
**Immune system problems (e.g., swollen lymph**	9	4	13
**glands, fever, fatigue)**	7.30%	4.00%	5.80%
**Gastrointestinal problems (e.g., nausea, bloating,**	8	6	14
**cramping, diarrhea)**	6.50%	6.10%	6.30%
**Cardiovascular problems (e.g., fast or irregular**	10	3	13
**heartbeat, jitteriness, chest discomfort)**	8.10%	3.00%	5.80%
**Musculoskeletal problems (e.g., muscle or joint**	9	2	11
**pain, cramps, weakness)**	7.30%	2.00%	4.90%
**Other**	3	5	8
	2.40%	5.10%	3.60%
**SUM**	280	158	438
	225.80%	159.60%	196.40%

Table 17

B2. Do any of these health problems mean a total or partial loss of bodily or mental functions, for you personally?

	Asthmatic	Non-Asthmatic	GenPop
**Total**	174	188	362
	100.00%	100.00%	100.00%
**Yes**	47	15	62
	27.00%	8.00%	17.10%
**No**	113	156	269
	64.90%	83.00%	74.30%
**Don't know/not sure**	14	17	31
	8.00%	9.00%	8.60%
**Decline to answer**	-	-	-
	-	-	-
**SUM**	174	188	362
	100.00%	100.00%	100.00%

Table 18

Q9. Has a doctor or health care professional ever told you that you have asthma or an asthma-like condition?

	Asthmatic	Non-Asthmatic	GenPop
**Total**	313	785	1098
	100.00%	100.00%	100.00%
**Yes - asthma**	176	-	176
	56.20%	-	16.00%
**Yes - asthma-like condition**	151	-	151
	48.20%	-	13.80%
**No**	-	740	740
	-	94.30%	67.40%
**Don't know/not sure**	-	43	43
	-	5.50%	3.90%
**Decline to answer**	-	2	2
	-	0.30%	0.20%
**SUM**	327	785	1112
	104.50%	100.00%	101.30%

Table 19

Have you ever been unable or reluctant to use the toilets in a public place, because of the presence of an air freshener, deodorizer, or scented product?

	Asthmatic	Non-Asthmatic	GenPop
**Total**	313	785	1098
	100.00%	100.00%	100.00%
**Yes**	67	60	127
	21.40%	7.60%	11.60%
**No**	235	685	920
	75.10%	87.30%	83.80%
**Neutral/not sure**	11	38	49
	3.50%	4.80%	4.50%
**Decline to answer**	-	2	2
	-	0.30%	0.20%
**SUM**	313	785	1098
	100.00%	100.00%	100.00%

Table 20

If you enter a business, and you smell air fresheners or some fragranced product, do you want to leave as quickly as possible?

	Asthmatic	Non-Asthmatic	GenPop
**Total**	313	785	1098
	100.00%	100.00%	100.00%
**Yes**	97	86	183
	31.00%	11.00%	16.70%
**No**	167	606	773
	53.40%	77.20%	70.40%
**Neutral/not sure**	49	92	141
	15.70%	11.70%	12.80%
**Decline to answer**	-	1	1
	-	0.10%	0.10%
**SUM**	313	785	1098
	100.00%	100.00%	100.00%

Table 21

Have you ever been unable or reluctant to wash your hands with soap in a public place, because you know or suspect that the soap is fragranced?

	Asthmatic	Non-Asthmatic	GenPop
**Total**	313	785	1098
	100.00%	100.00%	100.00%
**Yes**	65	48	113
	20.80%	6.10%	10.30%
**No**	232	679	911
	74.10%	86.50%	83.00%
**Neutral/not sure**	16	56	72
	5.10%	7.10%	6.60%
**Decline to answer**	-	2	2
	-	0.30%	0.20%
**SUM**	313	785	1098
	100.00%	100.00%	100.00%

Table 22

Are you aware that a “fragrance” in a product is typically a chemical mixture of several dozen to several hundred chemicals, mostly synthetic and derived

	Asthmatic	Non-Asthmatic	GenPop
**Total**	313	785	1098
	100.00%	100.00%	100.00%
**Yes**	154	292	446
	49.20%	37.20%	40.60%
**No**	132	386	518
	42.20%	49.20%	47.20%
**Don't know/not sure**	27	105	132
	8.60%	13.40%	12.00%
**Decline to answer**	-	2	2
	-	0.30%	0.20%
**SUM**	313	785	1098
	100.00%	100.00%	100.00%

Table 23

Are you aware that fragrance chemicals do not need to be fully disclosed on the product label or material safety data sheet?

	Asthmatic	Non-Asthmatic	GenPop
**Total**	313	785	1098
	100.00%	100.00%	100.00%
**Yes**	71	126	197
	22.70%	16.10%	17.90%
**No**	210	543	753
	67.10%	69.20%	68.60%
**Don't know/not sure**	32	113	145
	10.20%	14.40%	13.20%
**Decline to answer**	-	3	3
	-	0.40%	0.30%
**SUM**	313	785	1098
	100.00%	100.00%	100.00%

Table 24

Are you aware that fragranced products typically emit hazardous air pollutants such as formaldehyde?

	Asthmatic	Non-Asthmatic	GenPop
**Total**	313	785	1098
	100.00%	100.00%	100.00%
**Yes**	81	122	203
	25.90%	15.50%	18.50%
**No**	207	550	757
	66.10%	70.10%	68.90%
**Don't know/not sure**	24	110	134
	7.70%	14.00%	12.20%
**Decline to answer**	1	3	4
	0.30%	0.40%	0.40%
**SUM**	313	785	1098
	100.00%	100.00%	100.00%

Table 25

Are you aware that even so-called natural, green, and organic fragranced products typically emit hazardous air pollutants?

	Asthmatic	Non-Asthmatic	GenPop
**Total**	313	785	1098
	100.00%	100.00%	100.00%
**Yes**	68	96	164
	21.70%	12.20%	14.90%
**No**	220	589	809
	70.30%	75.00%	73.70%
**Don't know/not sure**	24	97	121
	7.70%	12.40%	11.00%
**Decline to answer**	1	3	4
	0.30%	0.40%	0.40%
**SUM**	313	785	1098
	100.00%	100.00%	100.00%

Table 26

If you knew that a fragranced product emitted hazardous air pollutants, would you still use it?

	Asthmatic	Non-Asthmatic	GenPop
**Total**	313	785	1098
	100.00%	100.00%	100.00%
**Yes**	47	97	144
	15.00%	12.40%	13.10%
**No**	188	430	618
	60.10%	54.80%	56.30%
**Don't know/not sure**	78	256	334
	24.90%	32.60%	30.40%
**Decline to answer**	-	2	2
	-	0.30%	0.20%
**SUM**	313	785	1098
	100.00%	100.00%	100.00%

Table 27

Have you ever been prevented from going to some place because you would be exposed to a fragrance product that would make you sick?

	Asthmatic	Non-Asthmatic	GenPop
**Total**	313	785	1098
	100.00%	100.00%	100.00%
**Yes**	100	65	165
	31.90%	8.30%	15.00%
**No**	188	644	832
	60.10%	82.00%	75.80%
**Don't know/not sure**	25	74	99
	8.00%	9.40%	9.00%
**Decline to answer**	-	2	2
	-	0.30%	0.20%
**SUM**	313	785	1098
	100.00%	100.00%	100.00%

Table 28

Has any exposure to fragranced products in your work environment caused you to become sick, lose work days, or lose a job?

	Asthmatic	Non-Asthmatic	GenPop
**Total**	313	785	1098
	100.00%	100.00%	100.00%
**Yes**	57	28	85
	18.20%	3.60%	7.70%
**No**	233	696	929
	74.40%	88.70%	84.60%
**Don't know/not sure**	23	58	81
	7.30%	7.40%	7.40%
**Decline to answer**	-	3	3
	-	0.40%	0.30%
**SUM**	313	785	1098
	100.00%	100.00%	100.00%

Table 29

Would you be supportive of a fragrance-free policy in the workplace?

	Asthmatic	Non-Asthmatic	GenPop
**Total**	313	785	1098
	100.00%	100.00%	100.00%
**Yes**	158	312	470
	50.50%	39.70%	42.80%
**No**	58	186	244
	18.50%	23.70%	22.20%
**Neutral/not sure**	97	284	381
	31.00%	36.20%	34.70%
**Decline to answer**	-	3	3
	-	0.40%	0.30%
**SUM**	313	785	1098
	100.00%	100.00%	100.00%

Table 30

Would you prefer that health care facilities and health care professionals be fragrance-free?

	Asthmatic	Non-Asthmatic	GenPop
**Total**	313	785	1098
	100.00%	100.00%	100.00%
**Yes**	157	317	474
	50.20%	40.40%	43.20%
**No**	78	199	277
	24.90%	25.40%	25.20%
**Neutral/not sure**	77	265	342
	24.60%	33.80%	31.10%
**Decline to answer**	1	4	5
	0.30%	0.50%	0.50%
**SUM**	313	785	1098
	100.00%	100.00%	100.00%

Table 31

Flying On An Airplane That Pumped / Did Not Pump Scented Air Throughout The Passenger Cabin, Which Would You Choose?

	Asthmatic	Non-Asthmatic	GenPop
**Total**	313	785	1098
	100.00%	100.00%	100.00%
**Airplane with scented air**	58	121	179
	18.50%	15.40%	16.30%
**Airplane without scented air**	196	438	634
	62.60%	55.80%	57.70%
**Neutral/not sure**	59	224	283
	18.80%	28.50%	25.80%
**Decline to answer**	-	2	2
	-	0.30%	0.20%
**SUM**	313	785	1098
	100.00%	100.00%	100.00%

Table 32

Staying In A Hotel With / Without Fragranced Air, Which Would You Choose?

	Asthmatic	Non-Asthmatic	GenPop
**Total**	313	785	1098
	100.00%	100.00%	100.00%
**Hotel with fragranced air**	73	176	249
	23.30%	22.40%	22.70%
**Hotel without fragranced air**	188	422	610
	60.10%	53.80%	55.60%
**Neutral/not sure**	52	185	237
	16.60%	23.60%	21.60%
**Decline to answer**	-	2	2
	-	0.30%	0.20%
**SUM**	313	785	1098
	100.00%	100.00%	100.00%

Table 33

What is your household annual income?

	Asthmatic	Non-Asthmatic	GenPop
**Total**	313	785	1098
	100.00%	100.00%	100.00%
**Less than $10,000 (10)**	12	30	42
	3.80%	3.80%	3.80%
**$10,000 - $49,999 (29.99)**	81	156	237
	25.90%	19.90%	21.60%
**$50,000 - $99,999 (74.99)**	97	278	375
	31.00%	35.40%	34.20%
**$100,000-$149,999 (124.99)**	54	152	206
	17.30%	19.40%	18.80%
**$150,000-$200,000 (175)**	21	60	81
	6.70%	7.60%	7.40%
**Over $200,000 (200)**	13	24	37
	4.20%	3.10%	3.40%
**Decline to answer**	35	85	120
	11.20%	10.80%	10.90%
**Mean (In Thousands)**	82.19	85.89	84.84
**Standard Deviation**	51.98	49.53	50.24
**Standard Error**	3.12	1.87	1.61
**SUM**	313	785	1098
	100.00%	100.00%	100.00%

Table 34

Q1. Who Answer “Yes” To One Or More Of These Options For Q1.

	Asthmatic	Non-Asthmatic	GenPop
**Total**	313	785	1098
	100.00%	100.00%	100.00%
**Yes (Net)**	310	766	1076
	99.00%	97.60%	98.00%
Air fresheners and deodorizers (e.g., sprays, solids, oils, disks)	220	513	733
	70.30%	65.40%	66.80%
Personal care products (e.g., soaps, hand sanitizer, lotions, deodorant, sunscreen, shampoos)	293	713	1006
	93.60%	90.80%	91.60%
Cleaning supplies (e.g., all-purpose cleaners, disinfectants, and dishwashing soap)	263	650	913
	84.00%	82.80%	83.20%
Laundry products (e.g., detergents, fabric softeners, dryer sheets)	273	653	926
	87.20%	83.20%	84.30%
Household products (e.g., scented candles, toilet paper, trash bags, baby products)	247	600	847
	78.90%	76.40%	77.10%
Fragrance (e.g., perfume, cologne, after-shave)	223	541	764
	71.20%	68.90%	69.60%
Other	5	20	25
	1.60%	2.50%	2.30%
**None**	3	19	22
	1.00%	2.40%	2.00%
**SUM**	1527	3709	5236
	487.90%	472.50%	476.90%

Table 35

Q1a. Who Answer “Yes” To One Or More Of These Options For Q1A.

	Asthmatic	Non-Asthmatic	GenPop
**Total**	313	785	1098
	100.00%	100.00%	100.00%
**Yes (Net)**	289	678	967
	92.30%	86.40%	88.10%
Air fresheners and deodorizers (e.g., sprays, solids, oils, disks)	174	384	558
	55.60%	48.90%	50.80%
Personal care products (e.g., soaps, hand sanitizer, lotions, deodorant, sunscreen, shampoos)	206	469	675
	65.80%	59.70%	61.50%
Cleaning supplies (e.g., all-purpose cleaners, disinfectants, and dishwashing soap)	169	388	557
	54.00%	49.40%	50.70%
Laundry products (e.g., detergents, fabric softeners, dryer sheets)	140	346	486
	44.70%	44.10%	44.30%
Household products (e.g., scented candles, toilet paper, trash bags, baby products)	162	383	545
	51.80%	48.80%	49.60%
Fragrance (e.g., perfume, cologne, after-shave)	237	507	744
	75.70%	64.60%	67.80%
Other	6	14	20
	1.90%	1.80%	1.80%
**None**	24	107	131
	7.70%	13.60%	11.90%
**SUM**	1118	2598	3716
	357.20%	331.00%	338.40%

Table 36

Q1/Q1a. Who Answer “Yes” To One Or More Of These Options For Q1/Q1A (own use, other's use).

	Asthmatic	Non-Asthmatic	GenPop
**Total**	313	785	1098
	100.00%	100.00%	100.00%
**Yes (Net)**	312	770	1082
	99.70%	98.10%	98.50%
Air fresheners and deodorizers (e.g., sprays, solids, oils, disks)	250	576	826
	79.90%	73.40%	75.20%
Personal care products (e.g., soaps, hand sanitizer, lotions, deodorant, sunscreen, shampoos)	302	730	1032
	96.50%	93.00%	94.00%
Cleaning supplies (e.g., all-purpose cleaners, disinfectants, and dishwashing soap)	280	684	964
	89.50%	87.10%	87.80%
Laundry products (e.g., detergents, fabric softeners, dryer sheets)	286	695	981
	91.40%	88.50%	89.30%
Household products (e.g., scented candles, toilet paper, trash bags, baby products)	265	635	900
	84.70%	80.90%	82.00%
Fragrance (e.g., perfume, cologne, after-shave)	275	628	903
	87.90%	80.00%	82.20%
Other	8	26	34
	2.60%	3.30%	3.10%
**None**	26	111	137
	8.30%	14.10%	12.50%
**SUM**	1692	4085	5777
	540.60%	520.40%	526.10%

Table 37

Q2/Q3/Q4/Q5/Q6. People Who Answer “Yes” To One Or More Of These Questions: Q2/Q3/Q4/Q5/Q6 (fragrance sensitive group).

	Asthmatic	Non-Asthmatic	GenPop
**Total**	313	785	1098
	100.00%	100.00%	100.00%
**Yes**	174	188	362
	55.60%	23.90%	33.00%

Table 38

BA-Q2/Q3/Q4/Q5/Q6. People who answer “Yes” to each type of health problem under BA for each of these questions Q2/Q3/Q4/Q5/Q6.

	Asthmatic	Non-Asthmatic	GenPop
**Total**	313	785	1098
	100.00%	100.00%	100.00%
**Migraine headaches**	53	57	110
	16.90%	7.30%	10.00%
**Asthma attacks**	75	8	83
	24.00%	1.00%	7.60%
**Neurological problems (e.g., dizziness, seizures,**	28	21	49
**head pain, fainting, loss of coordination)**	8.90%	2.70%	4.50%
**Respiratory problems (e.g., difficulty breathing,**	106	77	183
**coughing, shortness of breath)**	33.90%	9.80%	16.70%
**Skin problems (e.g., rashes, hives, red skin,**	52	52	104
**tingling skin, dermatitis)**	16.60%	6.60%	9.50%
**Cognitive problems (e.g., difficulties thinking,**	27	18	45
**concentrating, or remembering)**	8.60%	2.30%	4.10%
**Mucosal symptoms (e.g., watery or red eyes, nasal**	83	71	154
**congestion, sneezing)**	26.50%	9.00%	14.00%
**Immune system problems (e.g., swollen lymph**	24	12	36
**glands, fever, fatigue)**	7.70%	1.50%	3.30%
**Gastrointestinal problems (e.g., nausea, bloating,**	21	15	36
**cramping, diarrhea)**	6.70%	1.90%	3.30%
**Cardiovascular problems (e.g., fast or irregular**	21	12	33
**heartbeat, jitteriness, chest discomfort)**	6.70%	1.50%	3.00%
**Musculoskeletal problems (e.g., muscle or joint**	18	11	29
**pain, cramps, weakness)**	5.80%	1.40%	2.60%
**Other**	5	16	21
	1.60%	2.00%	1.90%

Table 39

Demographics.

	Asthmatic	Non-Asthmatic	GenPop
**Total**	313	785	1098
	100.00%	100.00%	100.00%
**Male/Female**			
**All Males**	143	400	543
	45.70%	51.00%	49.50%
**All Females**	170	385	555
	54.30%	49.00%	50.50%
**Gender vs Age**			
**Male 18-24**	16	54	70
	5.10%	6.90%	6.40%
**Male 25-34**	34	75	109
	10.90%	9.60%	9.90%
**Male 35-44**	33	86	119
	10.50%	11.00%	10.80%
**Male 45-54**	34	92	126
	10.90%	11.70%	11.50%
**Male 55-65**	26	93	119
	8.30%	11.80%	10.80%
**Female 18-24**	26	60	86
	8.30%	7.60%	7.80%
**Female 25-34**	35	95	130
	11.20%	12.10%	11.80%
**Female 35-44**	42	95	137
	13.40%	12.10%	12.50%
**Female 45-54**	41	74	115
	13.10%	9.40%	10.50%
**Female 55-65**	26	61	87
	8.30%	7.80%	7.90%
