# Supplementary material for: Fragranced consumer products: effects on asthmatic Australians
Source: Air Qual Atmos Health. 2018 Mar 17;11(4):365–71. doi: 10.1007/s11869-018-0560-x (PMC5954056; doi:10.1007/s11869-018-0560-x)
Supplement: Supplementary file 2 — (PDF 55 kb) [file 11869_2018_560_MOESM2_ESM.pdf]

Supplementary Material 1 - Survey Methodology

Following Eysenbach G, 2004, Improving the Quality of Web Surveys: The Checklist for Reporting Results of Internet E-Surveys (CHERRIES), Journal of Medical Internet Research, Jul-Sep; 6(3): e34.

	**Checklist for Reporting Results of Internet E-Surveys (CHERRIES)**	
Item Category	***Checklist Item***	***Explanation***
**Design**		
	Describe survey design	Target population: national random sample of adults (ages 18-65) in Australia, representative of age, gender, and region (n=1,098, confidence limit=95%, margin of error=3%). The survey drew upon participants from a large web-based panel (over 200,000 participants in Australia) held by Survey Sampling International (SSI). Participant recruitment followed a randomized process (Dynamix). All responses were anonymous. Survey completion time was approximately ten minutes.
**IRB (Institutional Review Board) approval and informed consent process**		
	IRB approval	Ethics approval was obtained by the University of Melbourne, School of Engineering Human Ethics Advisory Group, application 1646894, on May 9, 2016.
	Informed consent	Participants had already provided informed consent as part of the SSI web-based panel. For this survey, participants were given the following introduction: aims of the study; principal researcher name, affiliation, and contact information; human ethics approval; length of survey (less than 15 minutes to complete); assurance that participation is completely voluntary and that all data provided are confidential; assurance that participants can withdraw at any time; outputs of research (findings will be provided through journal articles and website, free of charge); university human ethics contact information; and a specific question to indicate consent: "If you would like to participate in this project, please click the next button to proceed ('Go To Survey')."
	Data protection	Selected survey software and servers were used to ensure data protection. No personal information was linked to the survey results. The dataset (without any identifying information) is kept on password protected computers.

**Development and pre-testing**		
	Development and testing	The survey instrument was a 35-item questionnaire, developed and tested over a two-year period, including cognitive testing with 10 individuals and piloting with over 100 individuals, before full implementation in June 2016. This article reports results from the sub-populations of asthmatics and non-asthmatics. Steinemann (2017) reports results from the general population.
**Recruitment process and description of the sample having access to the questionnaire**		The survey invitation is an open invitation, rather than a direct invite, to the pool of panelists available at the time. The pool is filtered to achieve a representative sample through a set of initial questions for basic demographic characteristics.
	Open survey versus closed survey	Closed survey (only SSI participants), general population, random sample nationally representative of demographics.
	Contact mode	The survey provider, SSI, provided an open invitation to potential participants. The survey targeted the general population rather than a specific cohort.
	Advertising the survey	An open invitation was issued to randomly selected members of the web-based panel. The survey was not advertised.
**Survey administration**		
	Web/E-mail	The survey was web-based, with multiple choice and open format answers. All responses were anonymous, and collected through the online survey platform and stored on local password protected servers.
	Context	SSI is a survey research company and online panel provider.
	Mandatory/voluntary	Voluntary. Prospective participants were randomly invited to the survey.
	Incentives	Respondents were provided incentives for their participation by the panel provider, SSI, with points that can be redeemed for money or reward programs.
	Time/Date	Data were collected within one week in June 2016.
	Randomization of items or questionnaires	To prevent biases in response, five sets of questions were randomized for their multiple-choice items.
	Adaptive questioning	Eight questions were conditionally displayed based on responses to other items.
	Number of Items	The survey contained 35 questions. Each page contained one question with multiple choice and open format response categories.
	Number of screens (pages)	Overall, 36 to 44 pages were presented (including the introductory page), depending on responses to conditional items.
	Completeness check	All questions were required to be completed. All questions provided non-response options such as "don't know/not sure" and "decline to answer." Only completed surveys were included for analysis.
	Review step	Respondents were allowed one attempt per question, once they click "next" to review the next question; they are not allowed to go back to the previous questions or answers.

**Response rates**		Survey response rate: 93%. 1,183 initial responses; Number of drop outs: 53; Number of screen outs: 32; Number of completes: 1,098; Panel size, over 200,000. Panel response rate at time of survey implementation: 10.26%.
	Unique site visitor	Each respondent goes through stringent verification of identity upon signing up on SSI panel (including name, contact details, and IP). Once opt-in process is completed, each respondent is tagged with unique panel ID.
	View rate (Ratio of unique survey visitors/unique site visitors)	not applicable
	Participation rate (Ratio of unique visitors who agreed to participate/unique first survey page visitors)	SSI respondents are invited to survey through general population random selection. Unique clicks or visitors to the first page of the survey can be those who complete survey, drop out, or screen out.
	Completion rate (Ratio of users who finished the survey/users who agreed to participate)	93% (1,183 initial, 53 drop out, 32 screen out, 1,098 complete).
**Preventing multiple entries from the same individual**		
	Cookies used	not used
	IP check	SSI programming software and sampling tool, Dynamix, controls the traffic and ensures unique entries. Using unique Panel ID and IP, each respondent can attempt the survey only once.
	Log file analysis	not used
	Registration	This is a closed survey for SSI respondents only. Survey invite is mailed specifically to the e-mail address used upon joining survey and verified.
**Analysis**		
	Handling of incomplete questionnaires	Only completed questionnaires were included in the final dataset for analysis.
	Questionnaires submitted with an atypical timestamp	Minimum survey completion time was 5 minutes; average was 10 minutes. A small number of respondents were omitted for completing the items too quickly.
	Statistical correction	All demographic subgroups obtained statistically valid numbers to ensure a national representativeness.
